# A Computational Model for Path Loss in Wireless Sensor Networks in Orchard Environments

**DOI:** 10.3390/s140305118

**Published:** 2014-03-12

**Authors:** Hristos T. Anastassiu, Stavros Vougioukas, Theodoros Fronimos, Christian Regen, Loukas Petrou, Manuela Zude, Jana Käthner

**Affiliations:** 1 Department of Informatics and Communications, Technological and Educational Institute of Central Macedonia at Serres, End of Magnisias Str., GR-62124 Serres, Greece; E-Mail: hristosa@teiser.gr; 2 Department of Biological and Agricultural Engineering, University of California, Davis, One Shields Avenue, CA 95616, USA; 3 Department of Electrical and Computer Engineering, Aristotle University of Thessaloniki, Thessaloniki Gr-54629, Greece; E-Mails: frontheo@teemail.gr (T.F.); loukas@agro.auth.gr (L.P.); 4 Leibniz Institute for Agricultural Engineering Potsdam-Bornim (ATB), Beuth University of Applied Sciences Berlin, Max-Eyth-Allee 100, D-14469 Potsdam-Bornim, Germany; E-Mails: cregen@atb-potsdam.de (C.R.); mzude@atb-potsdam.de (M.Z.); jkaethner@atb-potsdam.de (J.K.)

**Keywords:** electromagnetic waves, attenuation, range, foliage, canopy, agriculture

## Abstract

A computational model for radio wave propagation through tree orchards is presented. Trees are modeled as collections of branches, geometrically approximated by cylinders, whose dimensions are determined on the basis of measurements in a cherry orchard. Tree canopies are modeled as dielectric spheres of appropriate size. A single row of trees was modeled by creating copies of a representative tree model positioned on top of a rectangular, lossy dielectric slab that simulated the ground. The complete scattering model, including soil and trees, enhanced by periodicity conditions corresponding to the array, was characterized via a commercial computational software tool for simulating the wave propagation by means of the Finite Element Method. The attenuation of the simulated signal was compared to measurements taken in the cherry orchard, using two ZigBee receiver-transmitter modules. Near the top of the tree canopies (at 3 m), the predicted attenuation was close to the measured one—just slightly underestimated. However, at 1.5 m the solver underestimated the measured attenuation significantly, especially when leaves were present and, as distances grew longer. This suggests that the effects of scattering from neighboring tree rows need to be incorporated into the model. However, complex geometries result in ill conditioned linear systems that affect the solver's convergence.

## 1. Introduction

### 1.1. Wireless Sensor Networks in Orchards

Wireless sensor networks (WSNs) have started to gain ground in applications related to agriculture, originally for sensing and recently also for control purposes. Of particular interest are applications related to high value crops, such as fresh-market fruits grown in orchards that require high investment and intensive production measurements throughout the growing season. An important factor, which determines the effectiveness of a WSN, is the connectivity of its nodes, *i.e.*, the ability of each node to communicate with at least one of its neighbors, thus remaining connected to the network. Therefore, selecting the position and the level of transmission power of each node is a crucial task. It has been shown [1] that in areas of dense vegetation, such as orchards and forests, where the line of sight (LoS) between nodes is typically unavailable, tree foliage can cause additional attenuation, which affects the range and consequently the connectivity and stable functionality of a WSN.

In the past, several experiments have been conducted to quantify the behavior of radio signal propagation through forests [2–4]. Attempts have also been made to model the tree-induced attenuation of electromagnetic (EM) waves using analytical or empirical models. Analytical models typically oversimplify the geometry of the tree habitus in order to solve the wave propagation equations using standard mathematical techniques (exact or asymptotic). Empirical models assume a certain closed-form expression for signal attenuation and select model parameters based on dense field measurements. In this paper a different approach is proposed, which is based on the numerical solution of the equations describing the combined propagation and scattering mechanism. As a first step, a digital version of the geometry corresponding to a typical tree is created, based on actual measurements taken at the spot. Furthermore, the EM properties of all scatterers involved are estimated by measuring the water content of wood, foliage, soil, and utilizing existing bibliography. The resulting model combines geometric and physical characteristics of the environment under test, and a commercial software package capable of simulating EM wave propagation is employed.

### 1.2. Path Loss in Free Space and Plane Earth Models

For an EM wave propagating in free space, path loss can be calculated by the Friis equation [5], which assumes the absence of any obstacles nearby. When the transmitting and receiving antennas are located close to the ground, their corresponding heights should also be taken into account. Even in that case, theoretical models differ considerably from measurements. Therefore, the empirical Fitted Plane Earth model is often used, which heuristically corrects several coefficients involved in the mathematical expressions, using information obtained from actual measurements. Details of the plane earth models are included in the appendix.

### 1.3. Empirical Models of Path Loss through Foliage

The prediction of path loss in orchards is a complicated task. In addition to reflection from the ground, tree canopies, trunks, branches, and leaves cause diffraction and scattering of the radio wave. Empirical models are often used to predict path loss, in order to avoid complex analytical models that require knowledge of many parameters such as electromagnetic parameters, soil and leave moisture, geometric characteristics, *etc.* Empirical models require the existence of real data collected at the specific area, which are then used for fitting the model parameters. Such a model is the Modified Exponential Decay Model (MED) [6] that spans a frequency range from 230 MHz to 95 GHz. The MED model is based on measurements for the specific frequency range, collected from areas where the propagation path is blocked by leaved trees. It is considered to be more accurate for propagation through foliage than for propagation above the canopy. The loss predicted by the model should be added to the loss in free space or the loss calculated from plane earth models. Another empirical model is the Best-Fit Parametric Exponential Decay model (BFPED) [7], which is a parametric version of the MED model. In this model, parameters *A*, *B* and *C* of the MED model are calculated to result in the best fit between the MED model and real data measurements. The ITU_R model (ITU-R P.833-2) [8] preceded the MED model and is also based on extensive measurements in areas with vegetation for the same frequency range as in MED. A variant of the ITU model, *i.e.*, the Fitted-ITU [9] (FITU-R) makes a clear distinction between propagation predictions through leaved or bare trees.

Although focusing on the aforementioned models is not the purpose of this paper, comparison among measurements against data extracted through all of them will be provided at least for one particular experimental layout, to quantify their relative accuracy with respect to the method proposed herein. In particular, the measurements and the results of the computational model proposed in this paper will be compared against the predictions of the Free Space, Fitted PE, MED, BFPED, ITU-R, and FITU-R models.

### 1.4. Analytical Models of Path Loss through Foliage

Contrary to empirical models, analytical models require knowledge of a set of propagation-related parameters regarding the environment (e.g., tree geometries), the EM properties of the soil, tree-branches and leaves (e.g., permittivity, permeability and conductivity). A well-established analytical tool is the Radiative Energy Transfer Model (RET) [10]. According to [11] RET is considered to be highly effective for propagation through areas with vegetation. It can be applied to signals of frequency above 1 GHz and is adaptive to a variety of radio path geometries. The set of equations describing the model can be found in [12]. Evaluation of four input parameters is required, which can be achieved by signal strength measurements. The advantage of the RET model in comparison with the previous empirical models is that it takes into account the scattering components of the signal.

A generic model of 1–60 GHz narrowband radio signal attenuation in vegetation was suggested earlier [12,13]. Several propagation modes were accounted for, such as edge diffraction, ground reflection and direct (through vegetation). Each propagation component was modeled according to empirical or analytical models such as FITU-R and RET and the superposition of all produced the final outcome. For the evaluation of the various input parameters, extensive measurements at various locations with different tree species were necessary.

Other analytical approaches for modeling the EM wave propagation include the use of the Uniform Theory of Diffraction (UTD) [14], full wave analysis [15] or Physical Optics (PO) [16]. UTD was used to model propagation through a durian tree orchard, providing estimation close to real measurements [17]. However the effects of leaves and branches were not taken into consideration, since the average height of a durian tree was 10 m and the transmitters were placed at much lower heights. Tree trunks were modeled as impedance cylinders. A full wave analysis solution in conjunction with Monte Carlo simulation was suggested [18], in order to evaluate the statistics of the spatial and spectral behavior of the field at the receiver. In the theoretical model presented in [16], the tree canopies were represented by a partially absorbing phase screen, whose properties were found by calculating the mean field in the canopy of the tree. PO was then used to evaluate the diffracting field at the receiver level.

Propagation in plantations is also addressed in [19], in coniferous or deciduous forests [20], and in agricultural fields and gardens [21], where measurements were compared to the empirical models described above. In [22] additional frequency bands are treated for propagation in forests and meadows, whereas in [23] the effect of surface waves for communication paths adjacent to the ground is studied for flat and irregular terrains. Finally, a review of already established models for path loss in vegetation is presented in [24].

### 1.5. Objectives, Comparison with Past Work, and Paper Structure

In the present approach a scattering model of a row of trees was constructed and the radio wave propagation was analyzed via a commercial Finite Element Method (FEM) solver. For this purpose, the geometry of a ‘typical’ cherry tree in an actual orchard was digitized, and a simplified Computer Aided Design (CAD) model of the tree was constructed. Estimates of the electrical properties (*i.e.*, permittivity, permeability and conductivity) pertaining to air, soil and foliage were made based on water content measurements, according to well-established methods described in Section 2.3.2. Signal power measurements were taken in the cherry orchard and were compared against the solution computed by the FEM solver, and by the empirical models that were applied by the authors in [25].

The paper is organized as follows: in Section 2 material and methods are described, including tree geometry measurements, signal power measurements, and modeling through FEM. Details on the antenna characteristics, material properties and geometry discretization are provided. In Section 3, computational data are extracted and compared to measurements and analytical/empirical models. In Section 4 useful conclusions are drawn, whereas Section 5 serves as an Appendix containing some well-known mathematical expressions related to the plane earth model.

## 2. Materials and Methods

### 2.1. Tree Geometry Measurements

The orientations of the tree branches were measured using a 3D tilt sensor (InertiaCube3^TM^, Intersense, Billerica, MA, USA). The sensor provides three degrees of freedom (yaw, pitch, roll) and angular range of 360° in all axes. In order to create a geometrical representation of cherry trees, measurements of the orientation, length and perimeter of the trunk and the main branches of a single representative tree were taken. The orchard was a commercial one and trees were similar in shapes and size.

The sensor was placed initially near the base of the trunk, thus defining the origin of a local 3D coordinate system (*x*, *y*, *z*) where roll, pitch and yaw angles were set to zero. Thereafter, the sensor was placed at the midpoint of the trunk and the rest of the branches and the roll, pitch and yaw values that were measured each time were stored in a PC connected to the sensor.

Since the representation of the tree would be implemented by using cylinders for each branch, every branch that could not be considered as a unique cylinder was split into two or more shorter parts whose orientation, length and perimeter were measured separately. In total, thirty-seven tree parts were measured.

### 2.2. Signal Power Measurements

**Figure 1.**
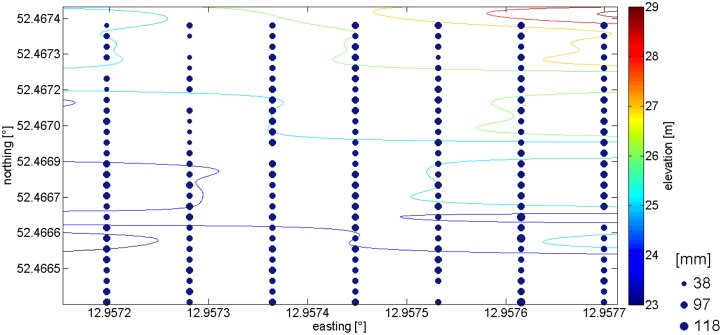
Layout of the cherry orchard.

The RF module used by the receiver and the transmitter nodes was the XBee-Pro^®^ ZB (Part Number: XK-Z11-M, Digi-Key Inc., Thief River Falls, MN, USA). This RF module implements the ZigBee protocol stack, meets the IEEE 802.15.4 standards and operates within the ISM 2.4 GHz frequency band. The transmitter power was 40 mW (+16 dBm) and the receiver sensitivity was −100 dBm (1% packet error rate) and both modules used monopole integrated whip antennas with a gain equal to *G* = 1.5 dBi (omnidirectional). The propagation performance was calculated based on the Receive Signal Strength Indicator (RSSI), which provides a measure of the strength of the RF signal in dBm (decibel-milli Watt) units. To estimate the RSSI at a receiver location, the transmitter was commanded to send 1,000 packets at an RF data rate of 250,000 bps. The mean value of all the RSSI measurements was reported as the RSSI of the specific location. The experiment of measuring the signal strength was split in two phases. The first phase took place between 27th and 31st of August 2012 while the cherry trees (*Prunus avium*) had leaves in a mature expanded stage. The second phase took place in late autumn between 12 and 16 of November 2012, when the trees were defoliated. The cherry orchard subjected to the measurements is located in the “Werderaner” cultivation area in Brandenburg, Germany. The investigated area captures 25 m by 110 m and is located on a hillside. The orchard (Figure 1) consists of 189 cherry trees arranged in 7 columns (left column is #1) and 27 rows (bottom row is #1).

The transmitter was placed 1 m away (outside the orchard) from the fourth tree of the first row; the transmitter power was set to 16 dBm. The receiver was placed inside the orchard, 1.5 m in front of every second tree, starting from the tree in the first row and first column. In total, the reception was measured at 107 trees. Each tree had a unique identification number and the numbering scheme is shown in Figure 1. The horizontal axis and the left vertical axis show the Universal Traverse Mercator (UTM) Easting and Northing coordinates in meters. The right vertical axis shows ground elevation in meters. The tree trunk diameters are displayed as black circles of varying diameters in millimeters. In all of the selected positions the receiver received a total of 1,000 radio packets at a radio rate of 250 kbps. Each packet was 128 bytes long, and was sent every 10 ms.

In both phases, three different scenarios were implemented. In each scenario the distance of the RF modules from the ground was different. In the first scenario the modules were placed 0.5 m above the ground, in the second 1.5 m above the ground, a height that is approximately near the middle of the foliage of every tree. Finally, in the third scenario, the modules were placed at a height of 3 m above the ground, which is very close to the average highest main branch of the tree.

### 2.3. Modeling of Radio Wave Propagation Using Finite Elements

#### 2.3.1. Models Employed

The EM propagation through a row of cherry trees in an orchard was modeled and simulated on the basis of the Finite Element Method (FEM) [26], using COMSOL Multiphysics*^®^*. This is a commercial package that can be applied to EM scattering problems. CAD models of trees were created based on measurements and were discretized in COMSOL via an unstructured mesh, by use of the tool's built-in mesh generator. To model the effect of the ground, the well-known four-path model [27] was originally considered as an efficient and accurate approximation. According to this approach, the total EM scattered field is a superposition of four components: (i) Field scattered directly by a tree; (ii) Field reflected from the ground and then scattered by a tree; (iii) Field scattered by a tree and then reflected from the ground; and (iv) field reflected from the ground, scattered by tree, and again reflected from the ground. However, to implement this model, values of both the amplitude and phase of the free-space fields were required, which are unavailable by COMSOL, which yields only the amplitudes of the electric and magnetic field. Therefore, the ground had to be modeled as a lossy dielectric slab. This approach unavoidably increased the number of Degrees of Freedom (DoFs) of the FEM model, and hence the size of the resulting algebraic linear system. For the working frequency of interest, and for the particular soil properties, the penetration depth of the EM field is only a few centimeters, meaning that the extra Degrees of Freedom (DoFs) could be kept down to a reasonable number. Finally, the geometry of a cherry tree row comprising 26 trees was encapsulated in a FEM ‘cage’, surrounded by Perfectly Matched Layers (PMLs), a feature that can also be activated almost automatically by COMSOL.

#### 2.3.2. Electromagnetic Simulation of the Antennas and the Environment

As stated before, the transmission and reception antennas in the measurement campaign were whip monopoles. It is well known from antenna theory [28] that the electric field produced by a dipole of length *l*, located along the *z*-axis and centered at zero, is given by:

(1) $$E_{\theta }=\frac{j\eta I_{m}}{2\pi r}e^{-\text{jkr}}\frac{\cos\left(\frac{kl}{2}\cos\theta \right)-\cos\left(\frac{kl}{2}\right)}{\sin\theta }$$

where *r* is the distance to the observation point, *θ* is the standard elevation spherical angle, *k* is the wavenumber, *η* is the vacuum impedance (377 Ω) and *I_m_* is the antenna current amplitude. In our case the length of the whip antenna was *λ*/2 (more precisely, it was a *λ*/4 monopole, located on a perfectly conducting/PEC surface). Moreover, it can be shown [28] that the total power radiated a *λ*/2 dipole is numerically equal to:

(2) $$P=36.57I_{m}^{2}\left(P\text{in W},I_{m}\text{in A}\right)$$

where the *λ*/4 monopole radiates exactly half as much. Given the fact that the transmitter power was equal to 40 mW, the current amplitude for the transmitter was easily found to be equal to *I_m_* = 0.047 A. The theoretical antenna gain is equal to 5.2 dBi, whereas the measured antenna gain was equal to 1.5 dBi, according to the antenna manual. The difference in the gain value is attributed to various losses, not taken into account in the theoretical modeling of the monopole. During the simulation, the transmitting antenna was positioned at three different heights, namely at 0.5, 1.5 and 3 m above the ground, and 1 m away from the first tree in the row, outside the orchard, just as in the measurement campaign.

The EM properties of the cherry tree wood could not be directly measured in the field. However, fairly accurate estimates of the permittivity and conductivity could be made, based on water content measurements. To begin with, wood exhibits negligible magnetic properties, meaning that the relative permeability was set equal to *μ_r_*=1. Moreover, extensive bibliography exists regarding the relative permittivity [29–31]. Based on field measurements, the average water content was found to be 10.9%. According to [29] typical values for the relative complex permittivity at 2.4 GHz for such water content values is approximately equal to *ε_r_* ≅ 3.5 − *j*0.6, almost the same for all directions, meaning that wood can safely be considered isotropic for the conditions of interest. From the imaginary part *ε*″ of the permittivity, one can easily calculate the conductivity *σ* of the wood. Indeed, *σ* = 2*πfε*_0_*ε″*, where *f* is the frequency and *ε*_0_ = 8.85 × 10^−12^ F/M. The corresponding value for the wood conductivity at 2.4 GHz is therefore easily calculated as *σ* ≅ 82mS/m.

The soil conductivity was directly measured at the field, and showed considerable variation with respect to the coordinates of the observation point. However, after repeated simulations, it was empirically shown that the exact value of the soil parameters did not affect significantly the EM path loss in the horizontal direction. Therefore, to simplify the simulation without significant loss of accuracy, an average value of *σ* ≅ 8mS/m was chosen for the conductivity; the real part of the relative permittivity was chosen as *ε′_r_* = 20 (‘Pastoral Hills, rich soil’ in [32]). For such a value of conductivity, the penetration depth *δ* is equal to:

(3) $$\delta =\sqrt{\frac{1}{\pi \mu_{0}f\sigma }}≅0.11\text{m}$$

where *μ*_0_ = 4*π* × 10^−7^H/m is the magnetic permeability in vacuum. This shallow penetration depth means that the soil can be simulated in COMSOL by a thin, lossy dielectric slab. To guarantee negligible propagation at a given depth, as well as acceptable behavior of the geometric mesh, the thickness of the slab was set to 2.5 m.

With respect to the foliage, again only estimates of the permittivity and conductivity could be made, based on the water content measurements that took place using the gravimetric method (weighing leaves before and after drying). The average water content was found to be 27.85%. According to [33] an empirical expression for the leaf relative complex permittivity as a function of the water content *Mg* is:

(4) $${\varepsilon^{\prime }}_{r}=3.95\exp(2.79\text{Mg})-2.25$$

(5) $${\varepsilon^{\prime \prime }}_{r}=2.69\text{exp}(2.15Mg)-2.68$$

Using the expressions above, along with the expression $\sigma =2\pi f\varepsilon_{0}{\varepsilon^{\prime \prime }}_{r}$it was found that the real part of the leaf permittivity was equal to ${\varepsilon^{\prime }}_{r}=6.37$ and the conductivity was equal to σ = 0.3 S/m.

These expressions have been verified for the X band, where the wavelength is on the order of 4 mm, whereas the wavelength in our application is 125 mm. An alternative model proposed in [34] valid for 1–100 GHz is expressed by:

(6) $$\varepsilon_{r}=0.689\left(Mg-0.24\right)\varepsilon_{SW}+4.35-3.84Mg$$

where *ε_SW_* is the permittivity of saline water, which is strongly dependent on frequency. For a frequency equal to *f* = 2 GHz, one obtains *ε_SW_* = 77−*j*22,and therefore, for *Mg* = 0.2785 the permittivity of the foliage becomes *ε_r_=*5.39−*j*0.6, *i.e.*, the conductivity is equal to σ = 0.008 S/m Therefore the values provided by the models in Equations (4)–(6) are of the same order.

### 2.4. Geometry Discretization

**Figure 2.**
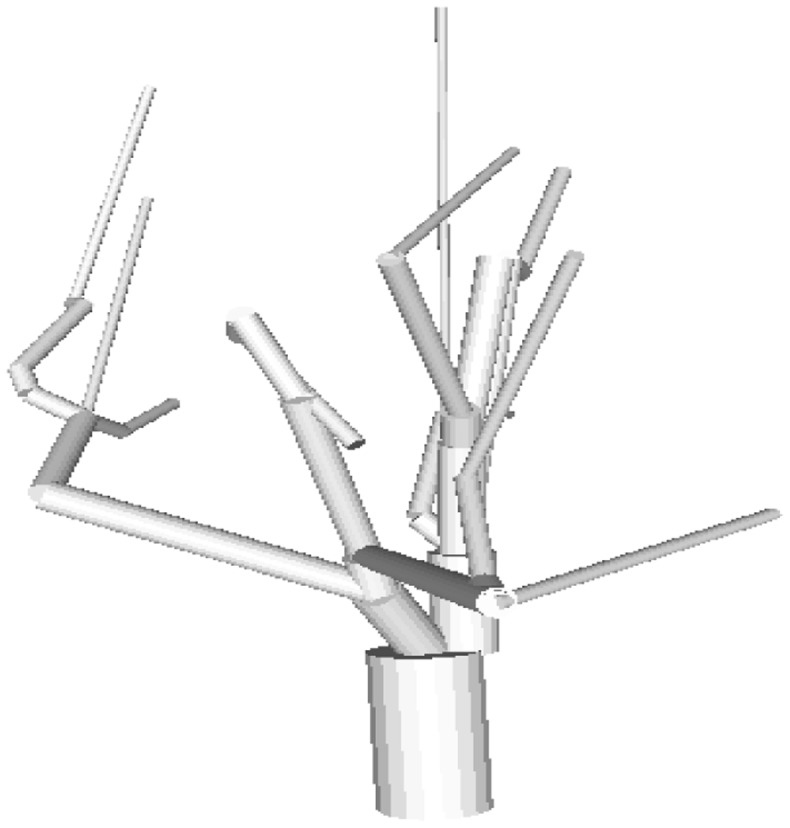
The complete CAD model of a single cherry tree (without leaves).

Originally, the entire CAD model (see Figure 2) was intended for meshing; however this procedure resulted in an overwhelming number of tetrahedra, whose size varied considerably. The immediate consequence of this situation was an exceedingly large linear system, even for a single tree; the size depended on the resolution of the mesh chosen through COMSOL. Invariably, in all cases the linear system proved to be highly ill conditioned, due to the coexistence of large and small elements, preventing all iterative solver algorithms in COMSOL from converging.

**Figure 3.**
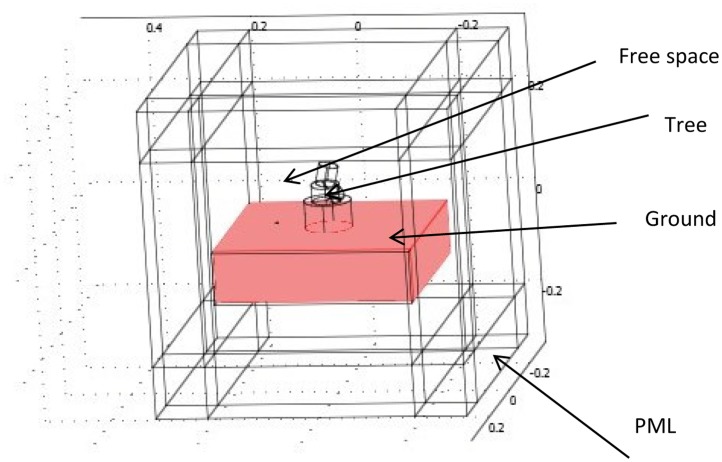
The simplified CAD model for a single cherry tree (no foliage).

To decrease the computational burden, only the main, thickest branches of the tree—including the trunk—were subsequently considered, (see Figure 3). Discretization of this simplified model was far more tractable, and EM scattering simulation through COMSOL became feasible.

Simulation of the foliage geometry is, in general, a very complicated task [34]. To be exact, each leaf needs to be simulated by a flat disk of appropriate shape, whereas the orientation and density have to be defined on the basis of probability theory. However, as already discussed for the branches, such a procedure is extremely expensive with respect to computational cost, and therefore a suitable approximation is absolutely essential. According to measurements and photos taken at the orchard, the canopy of a representative cherry tree—in that particular orchard—could be roughly approximated by a sphere of radius 1.5 m, centered 3 m over the ground. Therefore, the COMSOL model for the canopy was chosen as a dielectric sphere of the dimensions above, and the material properties as discussed earlier.

Next, the meshing and EM simulation of an entire row of cherry trees was attempted. To simulate the row, 27 copies of the simplified cherry tree model were located at 5 m separating distances, spanning a total of 130 m. The tree models were positioned on top of a rectangular, lossy dielectric slab, 2.5 m deep, to simulate the ground, as explained in the section before. Since in the measurement campaign the transmission direction was always chosen to be along a single row, the scattering effect of the remaining rows was considered negligible, and therefore the rows were not included in the simulation, to reduce the computational burden.

**Figure 4.**
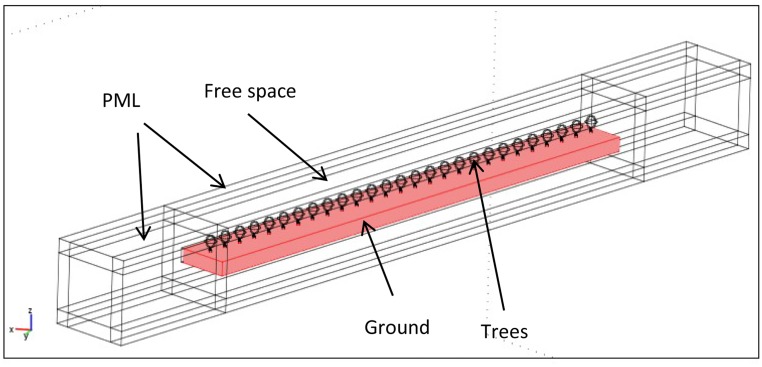
Row of cherry trees and soil slab embedded in a bounding box.

As discussed in the introduction, the array of 27 trees together with the slab simulating the ground was encapsulated in a rectangular box, since in the FEM the entire space surrounding the scattering geometry is theoretically discretized (see Figure 4). Of course, the mesh had to be truncated. The size of the box was chosen to be 30 m × 30 m × 150 m. The boundaries of the box were modeled by COMSOL as Perfectly Matched Layers (PMLs), absorbing the EM waves propagating perpendicularly to their surface. The mesh (when foliage was included) contained 53,836 elements, resulting in 342,370 Degrees of Freedom (DoFs). Without foliage, these numbers were 43,373 and 275,926 respectively. The algebraic linear system turned out again to be highly ill conditioned, and none of the iterative solvers integrated in COMSOL would converge. Numerical results could be extracted only through an out-of-core LU decomposition algorithm (PARADISO).

## 3. Results

**Figure 5.**
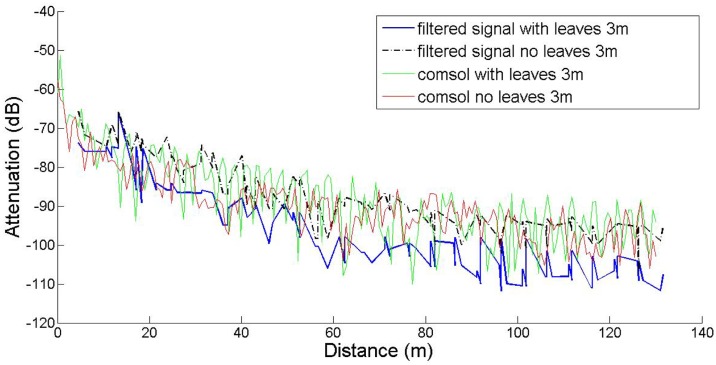
RSSI computations and measurements for transmitter and receiver height equal to *h* = 3.0 m.

**Figure 6.**
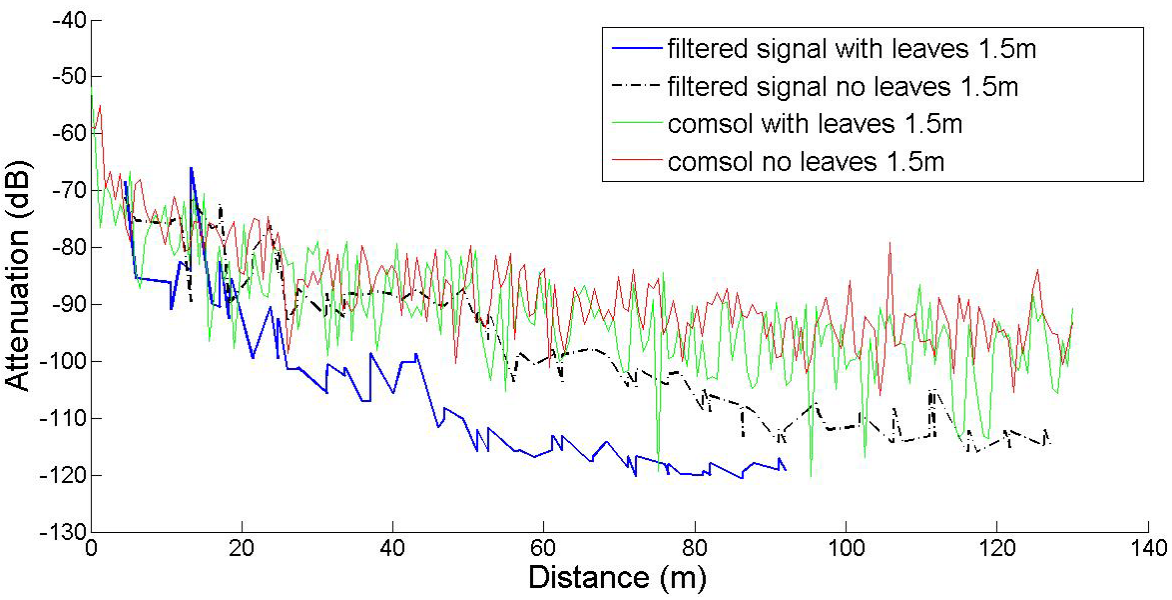
Attenuation computations and measurements for transmitter and receiver height equal to *h* = 1.5 m.

**Figure 7.**
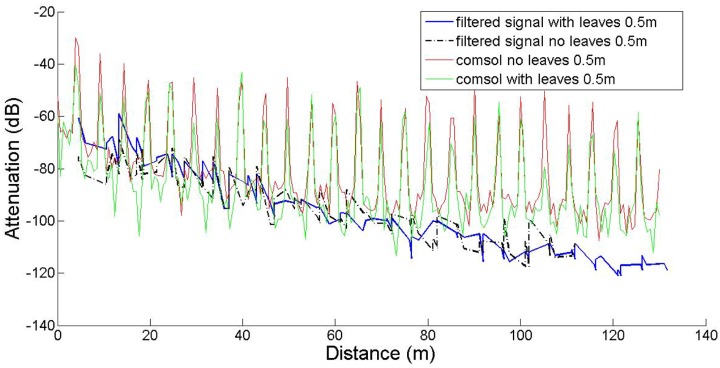
Attenuation computations and measurements for transmitter and receiver height equal to *h* = 0.5 m.

In this work our goal was to compare established empirical attenuation models, attenuation field measurements and the computational model developed with the *COMSOL* Multiphysics software package. Three sets of computations were performed, at three antenna heights, namely 0.5 m, 1.5 m and 3.0 m, with and without leaves, in accordance to the measurements. The comparisons between computed and measured data are shown in Figures 5–7. The signal attenuation (also known as path loss (*PL*)) in dB was calculated as in [25] by:

(7) $$PL=\left(P_{t}+G_{t}+G_{r}\right)-P_{r}$$

where *P_t,r_* and *G_t,r_* stand for the power and the gain of the transmitter (*t*) and receiver (*r*) respectively. For the measurement data both gains were assumed to be 1.5 dBi, whereas for the prediction data they were set equal to 5.2 dBi. The resulting data were processed according to [25]: they were first sorted according to increasing distance from the transmitter, and filtered with a 3rd order median filter to remove outliers. All computations took place on an Intel^®^ Pentium I-5 Quad Core processor running at 3.10 GHz with 8 GB RAM.

In the 1.5 m height case, the main lobe of the antenna pattern for the given antenna model is intersected by the scatterers (foliage, trunk) in a way similar to the 3 m case, resulting in analogous attenuation behavior. However, for the 0.5 m height case, the main lobe is significantly affected by the tree trunks, as demonstrated by the spikes of the corresponding plot, occurring exactly at the tree positions. The slight discrepancy between measured and predicted data may be partly attributed to inexact modeling of the actual antenna, due to the lack of crucial data in the antenna documentation.

The results indicate that the predictions and measurements curves resemble each other at 3 m, and at 1.5 m only in short distances and in the absence of leaves. However the predicted path loss at 1.5 m in the presence of leaves, especially at longer ranges is consistently lower. In the *h* = 0.5 m case, the narrow spikes correspond to the tree trunks, which behave like secondary transmitters, as expected. Measurements do not show this effect, since the receiver antenna was never located exactly on (or inside) any tree trunk. Moreover, the spikes are very narrow, meaning that even at very short distances away from the trunks, this local scattering effect becomes negligible.

**Figure 8.**
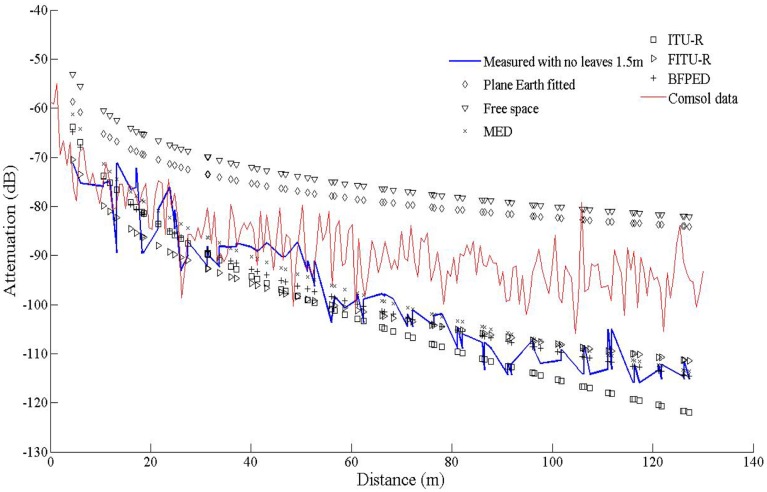
Comparison between empirical propagation models, measurements and data from simulation, for transmitter and receiver height at 1.5 m with no leaves.

**Figure 9.**
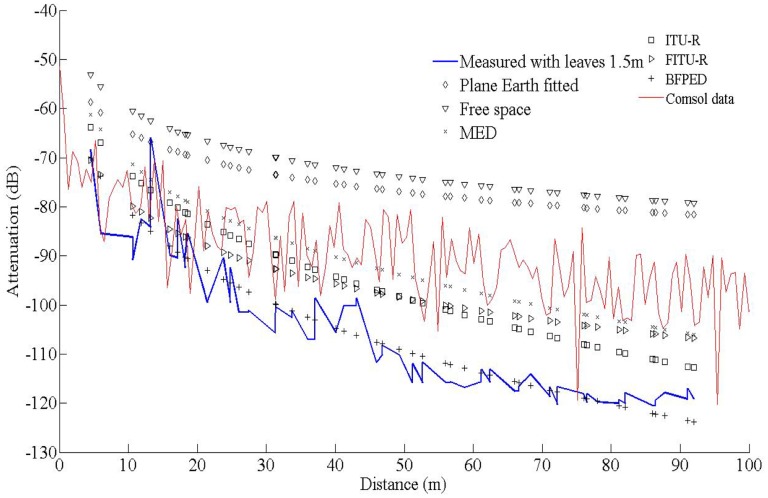
Comparison between empirical propagation models, measurements and data from simulation, for transmitter and receiver height at 1.5 m, with foliage.

At the height of 3 m the receiver and transmitter are always close to the canopy top and the presence of leaves does not seem to greatly affect the attenuation; the predicted attenuation is slightly smaller than the measured one. However, at 1.5 m the signal propagates through the center part of the canopies and foliage and branches affect a larger portion of its travel. This is the reason why the maximum communication range decreased from 130 m to 96 m, *i.e.*, the receiver could not receive packets any more. In former studies in plum orchards with smaller tree size this effect was marginal. The computational model did not capture this effect; instead it consistently predicted lower attenuation. This underestimation of the predictions against the measurements may be attributed to several factors. COMSOL provided only the amplitude and not the phase of the three components of the electric field. Therefore, all components were assumed to be coherent, and consequently the computed power at the receiver is an *upper bound* of the actual power; indeed, measurements tended to be lower than predictions overall. Also, any possible inadequate matching between antenna and transmission line would reduce the actual power captured by the receiver. The attenuation measurements, the predictions of the FEM solver, and the predictions of the empirical propagation models are shown in Figures 8 and 9, at a height of 1.5 m, with and without leaves respectively. In the absence of leaves, the BFPED and FITU-R models followed closely the measurements; BFPED requires extensive measurements in the orchard. The ITU-R model provided a more conservative ‘envelope’ for attenuation. In the presence of leaves only the empirical BFPED model was able to follow the measurements; this is of course not surprising, since its parameters were fitted to the measurement data. The computational model underestimated the attenuation in both cases, although in the presence of foliage the underestimation was more severe.

Finally, at longer distances from the antenna, neighboring tree rows start playing some role in the propagation, since they are increasingly ‘captured’ by the antenna lobe, as opposed to short distances, where they can safely be neglected. This observation explains partly the discrepancy in deterioration far away from the receiver. However, inclusion of neighboring rows increased the geometric complexity and resulted in an ill conditioned linear system that would not converge.

## 4. Conclusions and Outlook

In this paper a commercial Finite Element Method solver was used to calculate the electromagnetic propagation of radio waves between a pair of ZigBee nodes at 2.4 GHz, through a row of cherry trees. The model utilized an approximation of tree geometries, and estimates of the electrical characteristics of the air, soil and foliage based on water content measurements. Clearly, it would be impractical to digitize the exact geometry of all trees in a given orchard and use it in a FEM simulation. However, given the relative uniform training and pruning of trees in commercial orchards, a ‘representative’ tree could be defined, and used for all tree positions. Then, the number of tree columns and rows and their inter-spacing would suffice to estimate attenuation using an FEM solver. This paper constitutes a first step towards this goal.

The results showed that the computed solutions were in relatively good agreement with the measured attenuation curves and the empirical models near the treetops, where a large component of the signal is in free space. However, the FEM solver consistently underestimated the actual measured attenuation at a height corresponding to the middle of the canopies; the discrepancy was larger in the presence of leaves and at longer distances. To fully understand and explain the discrepancies, further research needs to take place. The effects of more precise geometric models and neighboring tree rows should be examined and their value should be weighed against the increased computational complexity. Accurate values for the real part of the permittivity of all scatterers are also essential; it is envisioned that libraries of tree geometric models and EM properties could be developed for this purpose, which is also of general interest in precision fruticulture. It is essential that the amplitude and the phase of the three components of the electric field be computed to account for the effects of wave scattering.

The significance of the results is not limited to a certain type of modulation. The overall methodology would be almost identical for any signal type (cellular, WLAN). The essential parameter that would need to be re-defined is the frequency of operation, meaning that the material properties should also be modified accordingly, based on experimental data obtained from the bibliography. Also, the transmitting and receiving antenna models should be taken care of, affecting obviously the radiation pattern.

Despite these difficulties, the proposed method promises to offer attenuation predictions that can be used for WSN deployment planning in an orchard, without the need for extensive RF measurements in the orchard, which are required by empirical models. This is essential, since obviously orchard grow and the WSN planning is certainly done only once for an orchard.

## Appendix

### Plane Earth Models

For an EM wave propagating in free space, path loss is described by the Friis equation:

(A1) $$PL_{fs}=G_{t}G_{r}\frac{\lambda^{2}}{{\left(4\pi d\right)}^{2}}$$

where *G_r_* and *G_t_* are the receiver and transmitter antenna gains, *d* is the distance between them and *λ* is the wavelength [5]. Therefore the signal power at the receiver *P_r_* is:

(A2) $$P_{r}=P_{t}PL_{fs}$$

where *P_t_* is the transmission power. However, it is more common to express path loss in dB using Equation (A3), which, combined with Equation (A1), results in Equation (A4):

(A3) $$PL_{fs}=10\log_{10}\frac{P_{t}}{P_{r}}$$

(A4) $$PL_{fs}=-27.56+20\log_{10}(f)+20\log_{10}(d)$$

where *f* is the signal frequency in MHz and *d* is the distance in m. In situations where the transmitting and receiving antennas are located close to the ground at heights *h_t_*, *h_r_* respectively, the above equations are not accurate enough, since the wave reflections off the ground should be taken into account. According to [5] the receiver power multiplied by the square of a factor $\frac{4\pi h_{r}h_{t}}{\lambda d}$ modifies the Friis equation into:

(A5) $$P_{r}≅P_{t}G_{t}G_{r}{\left(\frac{h_{r}h_{t}}{d^{2}}\right)}^{2}$$

This equation is named as the Plane Earth (PE) propagation model. The path loss for this model is calculated by:

(A6) $$PL_{PE}≅40\log_{10}d-10\log_{10}G_{t}-10\log_{10}G_{r}-20\log_{10}h_{t}-20\log_{10}h_{r}$$

In reality, soil is not perfectly conducting and the PE model results in large errors. To achieve modeling of higher fidelity, the empirical Fitted Plane Earth model has been used, which is a general mathematical expression of the following form:

(A7) $$PL_{PE-\text{fitted}}=A+B\log_{10}d-20C\left(\log_{10}h_{r}+\log_{10}h_{t}\right)$$

Calculation of *A*, *B* and *C* parameters is achieved by collecting signal strength measurements in free space with the antennas placed on heights *h_t_*, *h_r_*, and by minimizing the mean square error of the model with respect to the measurements. Further details on the aforementioned expressions can be found in [5] and [19].
